# Hazard control for communicable disease transport at Ornge

**DOI:** 10.1017/cem.2020.399

**Published:** 2020-05-21

**Authors:** Michael B. Peddle, Justin A. Smith

**Affiliations:** * Ornge, Mississauga, ON

**Keywords:** Aeromedical transport, communicable disease, COVID-19, hazard control, personal protective equipment

## Abstract

Transporting patients with communicable diseases is common in critical care transport operations. At Ornge, Ontario's critical care transport provider, 13.7% of patients required contact, droplet, or airborne precautions during transport in 2019–2020. Ensuring that staff are protected while transporting patients with communicable diseases must remain a prime directive for medical transport administrators and operators. Success in safety requires a robust system of hazard identification and adherence to generally accepted methods of hazard control. This commentary will discuss some of the administrative and engineering controls, as well as the personal protective equipment (PPE) strategies deployed at Ornge.

## INTRODUCTION

Transporting patients with communicable diseases is common in critical care transport operations. At Ornge, Ontario's critical care transport provider, 13.7% of patients required contact, droplet, or airborne precautions during transport in 2019–2020. Ensuring that staff are protected while transporting patients with communicable diseases must remain a prime directive for medical transport administrators and operators. Success in safety requires a robust system of hazard identification and adherence to generally accepted methods of hazard control. This commentary will discuss some of the administrative and engineering controls, as well as the personal protective equipment (PPE) strategies deployed at Ornge.

## ADMINISTRATIVE CONTROLS

In collaboration with infectious disease and Infection Prevention and Control (IP&C) experts, Ornge has developed the Infection Prevention and Control Resource Manual, which staff are required to review. The manual outlines Ornge's IP&C protocols, including a point-of-care risk assessment, hand hygiene, PPE, cleaning and disinfecting, health maintenance, and post-exposure protocols. Included in the manual is a table of “Infectious Diseases that Pose a Risk to Paramedics.” The table outlines disease signs and symptoms, modes of transmission, communicable period, recommended precautions, relative risk, and post-exposure follow-up for communicable diseases that a transport team may encounter.

Referring staff are required to provide specific details regarding suspected or confirmed communicable diseases in patients who require transport from one facility to another. Questions related to the level of precautions being observed at the referring facility are also documented in the electronic call record. The Transport Medicine Physician (TMP) reviews all requests for service, including the communicable disease screen and, in some cases, consults with the transport team before launch or during transit. The information gleaned from the screening process allows teams to pre-emptively prepare for a transport using the information contained in the IP&C document and through consultation with the TMP.

## ENGINEERING CONTROLS

Engineering controls include a broad category of barriers and processes designed to minimize the risk of communicable disease transmission. In hospital settings, a primary environmental engineering control is ventilation. Numerous organizations make recommendations around the required ventilation in hospital areas.^1^ Depending on the hospital area, the number of air changes per hour (ACH) recommended by the Centre for Disease Control (CDC) varies from 4–15.^2^ Barriers implemented in association with ventilation include the use of Airborne Infection Isolation Rooms (AIIRs), formerly called negative pressure isolation rooms. These spaces are designed to protect the staff in the area by exhausting contaminants, as well as staff and the environment outside of the room by ensuring unidirectional airflow. CDC guidelines require that AIIRs obtain a minimum of 6–12 total ACH with a negative pressure differential of -2.5 Pa.^2^

Instituting engineering controls in transport medicine is challenging. There exists a lack of information and recommendations related to environmental control in transport medicine. The CDC has provided guidance specific to severe acute respiratory syndrome (SARS) and Middle East respiratory syndrome coronavirus (MERS-CoV) transports.^3,4^ These include considerations of barriers when possible between the cockpit and cabin, as well as airflow that moves fore to aft. These recommendations do not consider the realities of air ambulance operations, nor do they reflect the realities of aircraft heating, ventilation, and air conditioning systems. Therefore, the CDC suggests that in aircraft with uncontrolled interior airflow, all crew should wear N95 or equivalent respirators during the transport of these patients. An in-depth investigation of this subject is needed.

Ornge uses the Pilatus PC-12 NG (PC12) and the Leonardo 139 (AW139) as their modes of fixed-wing and rotor-wing transport. The PC12 cockpit is not separate from the cabin. The interior cabin air exchange is approximately 10 ACH at baseline, but this varies depending on altitude, pressurization, and seal leakage. Interior airflow moves aft-to-front, and there is some cabin air recirculation. The AW139 has a highly variable air exchange rate and depends on the cabin fan speed selection. Aft cabin air exchange varies from 9 to 36 ACH with no recirculation. Ten of the 12 AW139 have a fixed solid barrier installed, allowing for independent cockpit and medical cabin ventilation. Land ambulances are capable of 24 ACH. The driver's compartment is separated from the patient compartment when a sliding pass-through window cover is closed. Understanding the impact of environmental engineering in vehicles allows organizations to appropriately assess risk exposure to staff and the PPE required for their crews during the transport of patients with communicable diseases.

## PERSONAL PROTECTIVE EQUIPMENT

In transport medicine, PPE is relied on heavily to protect the healthcare worker. Ensuring that frontline staff have appropriate PPE and robust PPE donning and doffing education is paramount. The Ornge IP&C manual outlines donning and doffing procedures for various levels of PPE. Additionally, training videos demonstrating PPE donning and doffing procedures are available to all our frontline staff to review safe donning and doffing procedures. Laminated pocket-sized and larger letter-sized donning and doffing checklists are mandated for use in every instance of PPE use.

The 2019 coronavirus disease (COVID-19) has put PPE supply chains in focus. Having an appropriate supply of PPE is critical to ensure safe frontline operations. A specific program to ensure adequate stock of all components of PPE is recommended. Building redundancy into any particular PPE plan is essential. Regular respiratory PPE fit testing is critical to provide logistics managers with timely and accurate information related to workforce equipment needs. Securing redundant supply chains for the various elements of PPE is an integral part of ensuring that all staff have the required PPE to do their job safely in the event of supply chain disruptions. Additionally, having policies, processes, and supply in place to use reusable PPE limit the impact of supply chain delivery disruptions because the items can be reused after appropriate cleaning or decontamination. Ornge is positioned to remove itself from the broader supply chain for 90–120 days during times of supply strain.

Recent supply chain constraints give credence to the concept of reusable PPE. Options that contemplate the uniqueness of the transport environment are recommended. Reusable half-face mask respirators with disposable filters can increase comfort for the staff member, especially for extended duration utilization. The introduction of this type of respiratory protection also reduces reliance on N95 type of respirators as the single method by which to provide respiratory protection.

The use of powered air-purifying respirators (PAPR) can be considered in certain circumstances. PAPRs provide enhanced respiratory protection compared with an N95, as well as potential improvements to staff comfort when working in PPE for extended periods or in warmer environments. However, there is no evidence to show that they reduce the risk of transmission of potentially airborne spread viral disease.^5^ Additionally, implementation requires consideration of numerous issues related to the transport environment and equipment specific to the organization. Special attention must be paid to the development of a thorough initial and continuing education program. Protocols specific to PAPR utilization when other PPE options may be appropriate are essential. Impacts on pilots and medical crew must also be considered when making organizational PPE choices. Limitations on the use of other safety equipment (i.e., helmets) need to be reviewed and carefully weighed before operational deployment. Ornge has not adopted the widespread use of PAPRs at the time of writing, although a robust training program with targeted information about self-contamination avoidance is currently in production.

**Figure 1.**
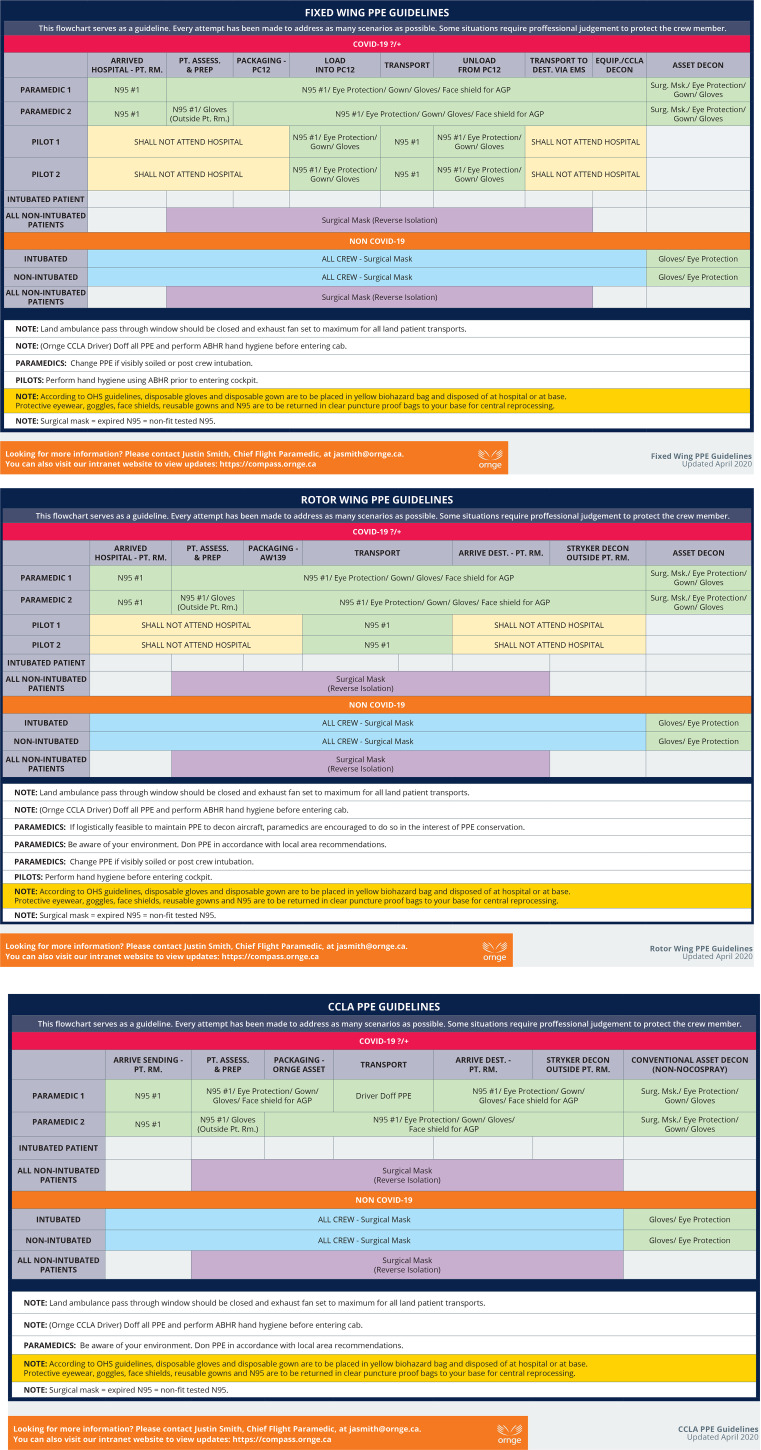
Personal protective equipment utilization flow chart.

Critical care medical transport via land or air comprises numerous phases that require differing levels of PPE for the various crew members. Having a clear understanding of what PPE is required in each phase is critical for crew safety and can minimize PPE burn by preventing overuse. Additionally, it can ensure that staff are comfortable that their level of PPE will keep them appropriately protected. Therefore, as an aid to staff, Ornge developed a PPE utilization flow chart for each of our asset types, outlining what PPE to don for each phase of transport (Figure 1). While these charts do not address every eventuality, they provide an overarching framework that frontline workers can refer to during times of increased cognitive load. The flow charts helped reduce staff anxiety around the transport of patients with communicable diseases knowing that the PPE they are in is appropriate for the asset and transport phase. A comprehensive and regular review of PPE recommendations on an ongoing basis, as well as additional scrutiny during epidemic and pandemic conditions, is essential in providing frontline staff confidence in their PPE.

## CONCLUSION

The transport of patients with communicable diseases is ubiquitous in medical transport. Organizations must have a vigorous IP&C program that considers the unique constraints of critical care medical transport on land and in air. The program should include a real-time system for hazard identification, as well as defined procedures that are responsive once a hazard is identified. An understanding of the specific environmental engineering controls related to their organizational assets is also recommended. An ongoing review of PPE supply, training, and education documents is an additional cornerstone of a robust IP&C program. Collectively, these elements will ensure that staff are both adequately protected but also comfortable and confident that they are safe when undertaking the care and transport of the critically ill patient with a communicable disease.

## References

[1] Public Health Agency of Canada. *Canadian tuberculosis standards 7th edition*; 2014 Available at: https://www.canada.ca/en/public-health/services/infectious-diseases/canadian-tuberculosis-standards-7th-edition.html (accessed April 18, 2020).

[2] CDC. Guidelines for environmental infection control in health-care facilities (2003); 2019 Available at: https://www.cdc.gov/infectioncontrol/guidelines/environmental/index.html (accessed May 3, 2020). 12836624

[3] CDC. Guidance on air medical transport for Middle East respiratory syndrome (MERS) patients; 2019 Available at: https://www.cdc.gov/coronavirus/mers/hcp/air-transport.html (accessed May 3, 2020).

[4] CDC. Guidance on air medical transport for SARS patients; 2004 Available at: https://www.cdc.gov/sars/travel/airtransport.html (accessed May 3, 2020).

[5] Wax RS, Christian MD. Practical recommendations for critical care and anesthesiology teams caring for novel coronavirus (2019-nCoV) patients. Can J Anesth 2020;67:568-76. 32052373 10.1007/s12630-020-01591-x PMC7091420

